# Genetic Variants in *PTGS1* and *NOS3* Genes Increase the Risk of Upper Gastrointestinal Bleeding: A Case–Control Study

**DOI:** 10.3389/fphar.2021.671835

**Published:** 2021-07-05

**Authors:** Marcela Forgerini, Gustavo Urbano, Tales Rubens de Nadai, Sabrina Setembre Batah, Alexandre Todorovic Fabro, Patrícia de Carvalho Mastroianni

**Affiliations:** ^1^ Department of Drugs and Medicines, School of Pharmaceutical Sciences, São Paulo State University (UNESP), Araraquara, Brazil; ^2^ Department of Surgery, School of Medicine, University of São Paulo, Ribeirão Preto, Brazil; ^3^ Department of Public Health, Bauru School of Dentistry, University of São Paulo (USP), Bauru, Brazil; ^4^ Department of Pathology and Legal Medicine, Ribeirão Preto Medical School, University of São Paulo, Ribeirão Preto, Brazil

**Keywords:** drug-related side effects and adverse reactions (MeSH D064420), genetic polymorphism, peptic ulcer disease, pharmacovigilance (MeSH), aspirin (MeSH)

## Abstract

**Objective:** To assess the association between *PTGS1* and *NOS3* variant alleles and the risk to develop upper gastrointestinal bleeding (UGIB) secondary to complicated peptic disease.

**Methods:** A case–control study was conducted in a Brazilian complex hospital from July 2016 to March 2020. *Case:* Patients with UGIB diagnosis. *Control:* Patients admitted for surgery not related to gastrointestinal disorders. Variables: UGIB (outcome), genetic variants in *PTGS1* and *NOS3* genes (independent), and sex, age, schooling, ethnicity, previous history of gastrointestinal disorders, *Helicobacter pylori* serology, comorbidity, drug therapy, and lifestyle (confounding). The single-nucleotide polymorphisms (SNPs) of the *PTSG1* gene (rs1330344, rs3842787, rs10306114, and rs5788) and *NOS3* gene (rs2070744 and rs1799983) were determined using the real-time polymerase chain reaction. *Helicobacter pylori* serology was determined through the chemiluminescence technique. Logistic regression models were built and deviations of allelic frequencies from *Hardy*–*Weinberg* equilibrium were verified.

**Results:** 200 cases and 706 controls were recruited. Carriers of the AG genotype of rs10306114 (OR: 2.55, CI 95%: 1.13–5.76) and CA + AA genotypes of rs5788 (OR: 2.53, CI 95%: 1.14–5.59) were associated with an increased risk for the UGIB development. In nonsteroidal anti-inflammatory drugs (NSAIDs) users, the six variants evaluated modified the magnitude of the risk of UGIB, whereas in low-dose aspirin (LDA) users, an increased risk of UGIB was observed for four of them (rs1330344, rs10306114, rs2070744, and rs1799983). Personal ulcer history (*p*-value: < 0.001); *Helicobacter pylori* infection (*p*-value: < 0.011); NSAIDs, LDA, and oral anticoagulant use (*p*-value: < 0.001); and alcohol intake (*p*-value: < 0.001) were also identified as independent risk factors for UGIB.

**Conclusion:** This study presents two unprecedented analyses within the scope of the UGIB (rs10306114 and rs2070744), and our findings showing an increased risk of UGIB in the presence of the genetic variants rs10306114 and rs5788, regardless of the drug exposure. Besides, the presence of the evaluated variants might modify the magnitude of the risk of UGIB in LDA/NSAIDs users. Therefore, our data suggest the need for a personalized therapy and drug use monitoring in order to promote patient safety.

## Introduction

Genetic variants in the *PTGS1* and *NOS3* genes may be important in the pathogenesis of UGIB, since these genes encode isozymes (e.g., *COX-1* and *COX-2*) and substances (e.g., nitric oxide) involved directly in several physiological processes in the platelet aggregation and gastrointestinal tract (Wallace and Miller, 2000; Brzozowski et al., 2005).

The main role of *COX-1* is platelet activation (Palma-Barqueros et al., 2020) and the production of prostaglandins through the arachidonic acid pathway. Prostaglandins, in turn, as well as nitric oxide, produced by the *NOS3* gene, mediate several gastric protection mechanisms, such as inhibition of gastric acid secretion and stimulation of mucus secretion and blood flow (Lanas, 2008; Agúndez et al., 2015).

The presence of genetic variants that affect the functionality of the *PTGS1* and *NOS3* genes may be clinically associated with a risk of bleeding (Lanas, 2008; Agúndez et al., 2015; Palma-Barqueros et al., 2020) and an increased sensitivity to certain drugs, such as low-dose aspirin (LDA) (Shiotani et al., 2014, 2015) and nonsteroidal anti-inflammatory drugs (NSAIDs) (Figueiras et al., 2016).

LDA is widely prescribed in the prevention and treatment of cardiovascular diseases due to its benefits and cost-effectiveness (Kochar et al., 2010), and a recently conducted meta-analysis suggests a protective effect of LDA against in some cancers (e.g., gastric, esophageal, and colorectal) (Qiao et al., 2018). In Brazil, it is estimated that 24.8% of population uses LDA for primary prevention of cardiovascular events and 34.3% for secondary (Vianna et al., 2012).

Despite the clinical benefits, LDA and NSAIDs have been reported as independent risk factors for upper gastrointestinal bleeding (UGIB) due to their gastro-erosive potential and for the irreversible inhibition of the *COX-1* and *COX-2* isozymes, which compromises the platelet aggregation cascade and increases bleeding risks (Lanas et al., 2015). Besides, a synergism in the potential for gastric damage and risk of UGIB was observed in LDA users with *Helicobacter pylori* infection (Huang et al., 2002) and/or carriers of genetic variants that modulate the platelet aggregation (Maree et al., 2005).

In this context, considering that UGIB is a digestive emergency associated with high morbidity (Quan et al., 2014) and seven out of every hundred patients admitted and underwent upper digestive endoscopy (UDE) in a tertiary hospital were diagnosed with UGIB (Forgerini et al., 2021c), studies have been assessing the influence of genetic variants on this risk, especially in LDA and NSAIDs users.

Through a recent systematic review (2020) (Forgerini et al., 2021a), three studies that evaluated single-nucleotide polymorphisms (SNPs) in the *PTGS1* and *NOS3* genes and the UGIB risk were identified (van Oijen et al., 2006; Wu et al., 2016; Mallah et al., 2020).

The first study was conducted by van Oijen et al. (2006), and the unique SNP (rs3842787) in the *PTGS1* gene assessed was not associated with the risk of UGIB (OR: 0.75, CI 95%: 0.19–3.01) (van Oijen et al., 2006). In 2016, Wu *et al.* evaluated this same SNP and two others (rs1330344 and rs3842788), and association data were only reported for the rs1330344 variant, which identified an increased risk of UGIB in homozygous patients (OR: 2.17, CI 95%: 1.01–4.66) (Wu et al., 2016). Finally, Mallah et al. (2020) evaluated three SNPs in the *PTGS1* gene (rs1330344, rs384287, and rs5788) and one SNP in the *NOS3* gene (rs1799983). The risk of UGIB was identified in LDA users and in the presence of CT + TT (rs1330344) (OR: 4.02, CI 95%: 1.11–14.54) and GT + TT genotypes (rs1799983) (OR: 7.690, CI 95%: 2.4–23.7) (Mallah et al., 2020).

Hence, to our knowledge, no study was conducted in the Brazilian population nor evaluated the SNPs rs10306114 in the *PTGS1* gene and rs2070744 in the *NOS3* gene (Forgerini et al., 2021a). Therefore, it was intended to evaluate the influence of those four SNPs already as described in the literature in addition to the two unpublished ones (rs10306114 and rs2070744) and the risk of UGIB.

## Methods

### Study Design, Setting, and Ethical Aspects

A case–control study, matched by sex, age (±5 years), and recruitment date (3 months), was conducted in the hospital complex of Clinical Hospital of the Ribeirão Preto Medical School of the University of São Paulo, Brazil.

This study was conducted in accordance with the Declaration of Helsinki and the Good Clinical Practice of the International Conference on Harmonization. Besides, the study was also registered in the Registro Brasileiro de Ensaios Clínicos (REBEC–number: RBR-3hstqm).

The report of this study was based on Strengthening the Reporting of Observational Studies in Epidemiology (STROBE) (von Elm et al., 2008).

### Participants

Participants were recruited for the case and control groups from July 2016 to March 2020. Considering the control group must be representative and recruited from the same “study base” as the case participants, participants of both groups were recruited from the same hospital complex (Setia, 2016).

#### Definition of Case and Control Groups

Patients admitted to the complex hospital with UGIB secondary to complicated peptic disease defined as the presence of endoscopically proven ulcers, bleeding erosions or perforated ulcers, and symptoms of hematemesis, dark vomiting or in coffee grounds, melena and/or hematochezia were included. Patients with excludable endoscopic diagnoses (e.g., esophageal varices, gastric neoplasia, and *Mallory–Weiss* tears) and patients with in-hospital UGIB were excluded. Secondary exclusion criteria included patients who did not undergo UDE within 48 h of hospital admission, discharge from hospital 15 days prior to the current admission, serious health condition, death, and those patients whose interview within the 15-day period preceding admission was not possible.

Being identified as a case, the patient was invited to participate in the study, and control participants were collected, matched by sex, age (±5 years), and hospital admission time (the maximum period of up to three months after the UDE date of the case participant). Control group participants were recruited from the preoperative unit of the same hospital complex, among patients who underwent surgery not related to gastrointestinal disorders (i.e., cataracts, inguinal/umbilical hernias, plastic, and prostate adenomas).

Patients under 18 years old; history of neoplasia, coagulopathies, immunodeficiency syndrome, and cirrhosis (history of disease); narcotics; carriers of a nasogastric or percutaneous tube; and nonresidents of the region of the study for at least three months were excluded.

### Data Collection and Variables

Four researchers (M.F, G.U, P.C.M, and T.R.N) conducted the recruitment of the participants in the case and control groups, using a questionnaire previous designed, validated (Figueiras et al., 2016), and adapted to be applied in Brazil. The data obtained during the interview with patients and/or family members were confirmed in secondary sources, such as the electronic medical record, medical prescriptions, and laboratory tests.

The interviewers evaluated the interviews on a scale from zero to 10, considering the reliability of the information recalled. The interview consisted of low reliability with scores below six points, and a reference value was adopted following the study by Figueiras et al. (2016).

**Dependent variable:** diagnosis of UGIB secondary to complicated peptic disease through UDE or exploratory laparotomy and clinical symptoms.

**Independent variable:** the presence of genetic variants in *PTGS1* and *NOS3* genes.

**Confounding variables:** sex, age, ethnicity, schooling, body mass index, previous personal history of gastrointestinal disorders (ulcer, dyspepsia, and bleeding), familiar history of ulcer, comorbidities (cardiovascular, blood, renal, kidney and respiratory diseases; high blood pressure; diabetes *mellitus*; dyslipidemia; depression; arthritis; and arthrosis), *Helicobacter pylori* infection, drug therapy in use (LDA, NSAIDs, oral anticoagulants, proton pump inhibitors, H2 receptors antagonists, and antidepressants), lifestyle habits (smoking, alcohol, and coffee consumption), and reliability of interviews.

An index date was defined as the day on which signs and symptoms of UGIB started for the cases and the date of the interview for the controls (Figueiras et al., 2016). To assess drug exposure, an etiological window of seven days prior to the index day was considered. Besides, LDA use was defined as the use of aspirin in doses lower than 150 mg and for a minimum period of three months.

The lifestyle habits in the two months preceding the index date were recorded. The smoking habit was stratified into nonsmoker, smoker and ex-smoker. For smokers, the amount of tobacco consumed was stratified as “moderate” (1–15 cigarettes/day) and “heavy” (>15 cigarettes/day).

Alcohol consumption in the two months prior to the index date was stratified into alcohol consumption and nonconsumption. Among the participants who consumed alcohol, the consumption was measured through the average amount in grams of alcohol consumed per day, being 0; >0 to ≤30 g of alcohol/day; and> 30 g of alcohol/day.

Smoking and alcohol consumption were stratified according the study of Figueiras et al. (2016).

Coffee consumption was stratified as consume or no consume. In those patients who consumed coffee, the consumption was categorized according to the frequency (daily, weekly, and monthly) and amount (mL = 0 ml; 0 < mL ≤ 100; and >100 ml).

### Collection of Biological Sample

After the interview, approximately 5.0 ml of whole blood was collected in ethylenediaminetetraacetic acid from the participants in the case and control groups for further analysis of the proposed genetic variants and the search for antibodies for *Helicobacter pylori* infection (IgG).

### Genotyping

The genomic DNA obtaining and quantification as well as genotyping were carried out at Centro de Medicina Genômica of Clinical Hospital of the Ribeirão Preto Medical School of the University of São Paulo and Laboratory of Pathology of the Ribeirão Preto Medical School of the University of São Paulo.

Genomic DNA extraction was carried out using the Maxwell® 16 Blood DNA Purification Kit (Promega, Madison, WI, United States) and the DNA quantification through the assay with Qubit ™ dsDNA HS Assay Kit (Applied Biosystems, Foster City, United States).

The genotyping technique was led in the StepOnePlus real-time PCR thermocycler (Applied Biosystems, Foster City, United States) using TaqMan Drug Metabolism Genotyping Assay technology (Applied Biosystems, Foster City, United States). For the *PTGS1* gene, the following assays were used: rs1330344 [C > T], assay ID C_31956972_10; rs10306114 [A > G], assay ID C_30090763_10; rs3842787 [C > T], assay ID C_30252576_20; rs5788 [C > A], and assay ID C_11722597_20. For the *NOS3* gene, the following assays were used: rs1799983 [G > T], assay ID C_3219460_20; and rs2070744 [C > T], assay ID C_15903863_10.

The real-time PCR conditions were performed with the following protocol: 1 min at 60°C, 10 min at 95°C, 40 cycles of denaturation for 15 s at 95°C, 40 cycles of annealing for 1 min at 60°C, and final extension for 1 min at 60°C. For quality control of genotyping, 10% of the samples were reanalyzed randomly.

### *Helicobacter pylori* Serology

The serology of *Helicobacter pylori* in order to determine IgG antibodies was performed on frozen plasma samples through the chemiluminescence technique. The test results were stratified into reagent (≥1.10 U IgG/mL), non-reagent (≤0.90 U IgG/mL), and inconclusive (0.91–1.09 U IgG/mL).

### Data Tabulation

The variables collected through the interviews were typed weekly in the Research Electronic Data Capture (REDCap) database, in addition to the results of genetic analyzes and laboratory tests for *Helicobacter pylori* infection.

### Statistical Analysis

The statistical distribution was tested using the Kolmogorov–Smirnov test, and considering that normality was identified, parametric statistical tests were used. Quantitative data were described through the mean and standard deviation, and qualitative data were described through absolute and relative frequency. Continuous variables were analyzed using the T-*Student* test and categorical variables using the chi-square test (BioEstat 5.0). Deviations of allelic frequencies from *Hardy*–*Weinberg* equilibrium were verified using the chi-square test (BioEstat 5.0).

The association between *PTGS1* and *NOS3* variant alleles and the risk to develop UGIB was estimated by odds ratio (OR) adopting a level of statistical significance of 0.05 for decision-making and building confidence interval with 95% (CI 95%) using unconditional logistic regression models (Kuo et al., 2018) in SPSS 20.0 software (IBM Company, Chicago, IL, United States).

The unconditional logistic regression models were designed for the dependent binomial variable (case or control), and participants with wild phenotype (the absence of genetic variant) and not users of LDA or NSAIDs were established as the reference category. Besides, considering the genetic variants, the participants were stratified in wild *vs.* heterozygous and homozygous genotype as well as wild *vs.* variant genotype (heterozygous + homozygous for variant allele).

A bivariate analysis was performed according to the outcome (UGIB) and all the predictive covariates of this study. From the bivariate analysis, variables with *p*-value ≤ 0.20 were selected for the logistic regression model. The regression model was built with all the predictive variables selected in the bivariate analysis using the methods of Insert (inclusion of all variables concomitantly).

In addition to the main logistic model, two other models were built. The first considered only LDA users and the second NSAIDs users. Therefore, the influence of the presence of genetic variants in these two subgroups was assessed.

### Data Transparency

This study is part of a larger study that aims to assess genetic variants associated with the risk of UGIB secondary to complicated peptic ulcer in the Brazilian population. The full-methodology protocol is available at Open Science Framework (OSF) (Forgerini et al., 2021b).

## Results

Two thousand eight hundred eighty-three UDE tests were conducted for 2,557 patients admitted to the complex hospital of Clinical Hospital of the Ribeirão Preto Medical School of the University of São Paulo.

As primary exclusion (*n* = 2,268), patients under 18 years old (*n* = 194), excludable endoscopic diagnoses (*n* = 1,828), in-hospital UGIB (*n* = 91), admission not due to UGIB (*n* = 79), those with a history of disease (*n* = 70), carriers of a nasogastric or percutaneous tube (*n* = 4), and nonresidents of the region of the study for at least three months (*n* = 2) were excluded. Secondary exclusion criteria included (*n* = 109) patients whose interview within the 15-day period preceding admission was not possible (*n* = 55), patients who did not undergo UDE within 48 h of hospital admission (*n* = 30), death (*n* = 9), discharge from hospital 15 days prior to the current admission (*n* = 8), and serious health condition (*n* = 7).

Therefore, through the daily monitoring and analysis of the UDE conducted during the period of this study, 180 patients were recruited for the case group. Besides, another 20 patients were also included after hospital admission for perforated gastric or duodenal ulcer and later endoscopic analysis (ICD10 K251 and K261). Thus, 200 patients were recruited to the case group and were paired with 706 controls.

Considering the epidemiological profile of the participants, most were men (case group: 72.5% and control group: 72.5%), self-declared white (case group: 67.3% and control group: 73.5%), and with a mean age of 60.1 (±16.3) and 59.9 (±15.8) years, respectively. Most of the patients were reagent for *Helicobacter pylori* infection (case group: 76.3% and control group: 57.6%), noncurrent smokers (case group: 70.1% and control group: 84.7%), abstain (case group: 51.5% and control group: 55.5%), and consumed coffee (case group: 85.0% and control group: 90.8%).

No interview received a score lower than six points, and most received scores higher than eight points (54.5% in the case group and 68.4% in the control group). The average of the interview scores was 7.60 (standard deviation: ± 1.26) in the case group and 8.27 (standard deviation: ± 1.36) in the control group. The baseline characteristics of the participants are described in Table 1.

**TABLE 1:** Baseline characteristics of the participants of case (*n* = 200) and control (*n* = 706) groups.

Variable	Case (%)	Control (%)	*p*-value^a^
	*n* = 200	*n* = 706	
Sex (men)	145 (72.5)	512 (72.5)	0.995
Mean age (±SD)	60.2 (±16.3)	59.8 (±15.8)	0.750^b^
Mean schooling (±SD)	6.3 (±4.3)	6.2 (±4.6)	0.750
**Ethnicity**			0.091^c^ ^,^ ^d^
White	134 (67.0)	516 (73.1)	
Black	65 (32.5)	186 (26.3)	
Oriental	1 (0.5)	4 (0.6)	
**Body mass index (kg/m^2^)**			0.003^d^
Underweight	10 (5.1)	15 (2.1)	
Normal weight	80 (40.4)	197 (28.0)	
Overweight	60 (30.3)	263 (37.4)	
Obesity I	30 (15.2)	152 (21.6)	
Obesity II	13 (6.6)	55 (7.8)	
Obesity III	5 (2.5)	21 (3.0)	
Missing data	2	3	
**Reliability of the interview**			<0.001^d^
6–7	91 (45.5)	223 (31.6)	
8–9	77 (38.5)	32 (45.9)	
>9	32 (16.0)	159 (22.5)	
**Previous history of gastrointestinal disorders**			
Familiar history of ulcer	41 (21.8)	132 (22.3)	0.870
Personal history of dyspepsia	60 (30.2)	291 (41.2)	0.004^d^
Personal history of ulcer	44 (22.1)	64 (9.1)	<0.001^d^
Personal history of bleeding	35 (17.6)	94 (13.3)	0.135^d^
***Helicobacter pylori*** ** serology**			<0.001^d^
Reagent	142 (76.3)	388 (57.6)	
No reagent	44 (23.7)	286 (42.4)	
Plasma sample not satisfactory	14	32	
**Comorbidity**			
Cardiovascular disease	62 (31.5)	131 (19.1)	<0.001^d^
Blood disease	3 (1.5)	42 (6.0)	0.009^d^
Renal disease	7 (3.5)	36 (5.2)	0.348
Respiratory disease	8 (4.0)	71 (10.1)	0.007^d^
High blood pressure	104 (52.0)	371 (52.5)	0.891
Diabetes *mellitus*	38 (19.1)	158 (22.4)	0.306
Dyslipidemia	21 (10.7)	165 (23.4)	<0.001^d^
Depression	20 (10.1)	81 (11.5)	0.559
Arthrosis	9 (4.5)	48 (6.9)	0.237
Arthritis	3 (1.5)	21 (3.0)	0.3218
**Drug therapy in use (ATC)**			
Proton pump inhibitors (A02BC)	36 (18.0)	125 (17.7)	0.923
H2 receptors antagonists (A02BA)	03 (1.5)	10 (1.4)	0.930
Oral anticoagulants (B01A)	22 (11.0)	18 (2.5)	<0.001^d^
Low-dose aspirin (B01AC06)	51 (25.5)	90 (12.7)	<0.001^d^
Nonsteroidal anti-inflammatory drugs (M01A)	43 (23.6)	71 (10.2)	<0.001^d^
Antidepressants (N06A)	19 (9.5)	70 (9.9)	0.962
**Lifestyle**			
Smoking habit			<0.001^d^
Nonsmoker	136 (70.1)	580 (84.7)	
Moderate (1–15 cigarettes/day)	23 (11.9)	52 (7.6)	
Heavy (>15 cigarettes/day)	35 (18.0)	53 (7.7)	
**Alcohol intake (average of grams of alcohol/day)**			<0.001^d^
Abstain (0 g)	103 (51.5)	392 (55.5)	
Little (0 > consume ≤30 g of alcohol/day)	71 (35.5)	297 (42.1)	
Moderate (>30 g of alcohol/day)	26 (13.0)	17 (2.4)	
Missing data	1	5	
**Coffee consumption**			0.260
No	29 (14.5)	65 (9.2)	
Yes	171 (85.5)	641 (90.8)	
Daily coffee consumption	159 (79.5)	599 (84.8)	
Weekly coffee consumption	10 (5.0)	35 (5.0)	
Monthly coffee consumption	2 (1.0)	6 (0.8)	
**Amount of coffee consumption per day (ml)**			<0.001^d^
mL = 0	30 (15.0)	66 (9.3)	
0 < mL ≤ 100	101 (50.5)	469 (66.4)	
mL > 100	69 (314.5)	171 (24.2)	

a *p-value* is polychotomous and represents the entire variable.

b *p-value* obtained in the T*-Student* test. The other *p*-values were obtained using the chi-square test.

c Oriental ethnicity was not considered for the chi-square test (number of participants <5) in these categories.

d Variables with *p*-value ≤ 0.20 and selected for the multivariate model.

ATC, Anatomical Therapeutic Chemical; SD, standard deviation.

The *Hardy–Weinberg* equilibrium was observed in both groups (case and control) for the six SNPs assessed, and the frequency of genetic variants in *PTGS1* and *NOS3* genes is described in Table 2.

**TABLE 2:** Description of frequency of genetic variants in *PTGS1* and *NOS3* genes between the case and control groups and *Hardy–Weinberg* equilibrium.

Variable	Case (%)	Control (%)	*p*-value^a^
	*n* = 200	*n* = 706	
**PTGS1 gene**			
**rs1330344 [C > T]**			0.095
CC (*wild type*)	28 (14.2)	69 (9.8)	
CT	82 (41.6)	274 (38.9)	
TT	87 (44.2)	362 (51.3)	
CT + TT	169 (85.8)	636 (90. 2)	
HWE (*p*-value)	0.2302	0.1088	
**rs10306114 [A > G]**			0.011*
AA (*wild type*)	161 (83.9)	631 (91.2)	
AG	30 (15.6)	60 (8.7)	
GG	1 (0.5)	1 (0.1)	
AG + GG	31 (16.1)	61 (8.8)	
HWE (*p*-value)	0.7528	0.7298	
**rs3842787 [C > T]**			0.699
CC (*wild type*)	157 (79.3)	577 (81.8)	
CT	40 (20.2)	124 (17.6)	
TT	1 (0.5)	4 (0.6)	
CT + TT	41 (20.7)	128 (18.2)	
HWE (*p*-value)	0.3576	0.3335	
**rs5788 [C > A]**			0.098
CC (*wild type*)	102 (52.3)	422 (60.4)	
CA	76 (39.0)	235 (33.6)	
AA	17 (8.7)	42 (6.0)	
CA + AA	93 (47.7)	277 (39.6)	
HWE (*p*-value)	0.5989	0.2287	
***NOS3* gene**			
**rs2070744 [C > T]**			0.907
CC (*wild type*)	38 (19.5)	141 (20.2)	
CT	88 (45.1)	321 (46.1)	
TT	69 (35.4)	235 (33.7)	
CT + TT	157 (80.5)	556 (79.8)	
HWE (*p*-value)	0.3012	0.1025	
**rs1799983 [G > T]**			0.414
GG (*wild type*)	92 (47.4)	359 (51.4)	
GT	87 (44.8)	277 (39.6)	
TT	15 (7.7)	63 (9.0)	
GT + TT	102 (52.5)	340 (48.6)	
HWE (*p*-value)	0.3681	0.3650	

a *p-value* obtained in the *chi-square test*.

HWE, *Hardy–Weinberg* equilibrium.

Regardless of LDA and NSAIDs use, an increased risk of UGIB was observed in carriers of AG genotype *vs.* AA of rs10306114 (OR: 2.55, CI 95%: 1.13–5.76) and in carriers of CA + AA genotypes *vs.* CC of rs5788 (*PTGS1* gene) (OR: 2.53, CI 95%: 1.14–5.59) (Table 3).

**TABLE 3:** Risk of upper gastrointestinal bleeding secondary to complicated peptic disease after the logistic regression model.

Variable	Logistic regression model		
	Insert method		
	OR	CI 95%	*p*-value
Constant	-	-	0.034
Ethnicity (self-declared black)	1.21	0.71–2.05	0.468
Ethnicity (self-declared oriental)	4.41	0.40–47.93	0.222
Body mass index (underweight)	1.20	0.34–4.25	0.771
Body mass index (overweight)	0.54	0.33–0.89	0.017^a^
Personal history of dyspepsia	0.40	0.24–0.69	<0.001^a^
Personal history of ulcer	3.82	1.88–7.75	<0.001^a^
Personal history of bleeding	1.59	0.82–3.10	0.166
*Helicobacter pylori* (reagent)	1.91	1.16–3.16	0.011^a^
Cardiovascular disease	1.54	0.82–2.88	0.177
Blood disease	0.14	0.02–0.81	0.028^a^
Respiratory disease	0.18	0.05–0.57	0.003^a^
Dyslipidemia	0.21	0.10–0.46	<0.001^a^
Oral anticoagulants use	10.58	3.58–31.25	<0.001^a^
LDA use	3.70	1.88–7.27	<0.001^a^
NSAIDs use	4.66	2.57–8.43	<0.001^a^
Smoking habit (1–15 cigarettes/day)	1.61	0.75–3.43	0.213
Smoking habit (>15 cigarettes/day)	1.97	0.97–3.87	0.058
Alcohol intake (0 < consume ≤30 g of alcohol/day)	2.58	1.41–4.74	0.002^a^
Alcohol intake (>30 g of alcohol/day)	7.98	2.98–21.32	<0.001^a^
Coffee consumption (0 < mL ≤ 100 ml)	0.48	0.23–1.00	0.051
Coffee consumption (>100 ml)	1.02	0.46–2.23	0.963
Reliability of the interview (scores 8–9)	0.57	0.34–0.95	0.030^a^
Reliability of the interview (score 10)	0.65	0.33–1.27	0.210
rs1330344 (CT genotype)	0.13	0.67–1.89	0.632
rs1330344 (TT genotype)	1.29	0.62–2.62	0.483
rs1330344 (CT + TT genotypes)	1.14	0.64–2.00	0.650
rs10306114 (AG genotype)	2.55	1.13–5.764	0.024^a^
rs10306114 (GG genotype)	-	-	-^b^
rs10306114 (AG + GG genotypes)	0.63	0.31–1.30	0.219
rs3842787 (CT genotype)	1.13	0.62–1.89	0.632
rs3842787 (TT genotype)	1.29	0.67–2.69	0.483
rs3842787 (CT + TT genotypes)	0.63	0.31–1.30	0.219
rs5788 (CA genotype)	1.00	0.61–1.63	0.990
rs5788 (AA genotype)	0.72	0.28–1.81	0.488
rs5788 (CA + AA genotypes)	2.53	1.14–5.59	0.022^a^
rs2070744 (CT genotype)	0.76	0.46–1.25	0.287
rs2070744 (TT genotype)	0.81	0.40–1.63	0.566
rs2070744 (CT + TT genotypes)	0.75	0.47–1.19	0.227
rs1799983 (GT genotype)	0.91	0.54–1.54	0.732
rs1799983 (TT genotype)	1.04	0.58–2.07	0.760
rs1799983 (GT + TT genotypes)	0.99	0.59–1.53	0.385

OR, odds ratio; CI 95%, confidence interval 95%; LDA, low-dose aspirin; NSAIDs, nonsteroidal anti-inflammatory drugs.

a Statistical significance.

b It was not possible to conduct the analysis due to the small number of participants in each group (one case and one control).

The category of reference for genetic variants is the wild phenotype.

Considering the *PTGS1* gene, LDA users showed an UGIB risk about four times higher in those carriers of the CT + TT genotypes *vs.* CC of rs1330344 (OR: 3.73, CI 95%: 2.00–6.95) and carriers of the AG + GG *vs.* AA of rs10306114 (OR: 4.15, CI 95%: 1.28–13.49). In NSAIDs users, the four variants evaluated were associated with the risk of UGIB (rs1330344, rs3842787, rs10306114, and rs5788), and this risk ranged from 2.71 in carriers of the CA + AA genotypes *vs.* CC of rs5788 (CI 95%: 1.251–5.88) to 5.69 in carriers of the AG + GG genotypes *vs.* AA of rs10306114 (CI 95%: 1.46–22.07) (Table 4).

**TABLE 4:** Logistic regression model for genetic variants in *PTGS1* and *NOS3* genes in low-dose aspirin and nonsteroidal anti-inflammatory drug users, and risk of upper gastrointestinal bleeding secondary to complicated peptic disease.

Variable	Logistic regression model			
	N case/control	Insert method		
		OR	CI 95%	p-value
LDA users^a^				
*PTGS1* gene				
rs1330344 (C > T)				
CT	22/31	5.76	2.50–13.23	<0.001^b^
TT	26/52	2.71	1.27–5.75	0.009^b^
CT + TT genotypes	48/83	3.73	2.00–6.95	<0.001^b^
rs10306114 (A > G)				
AG	8/10	3.95	1.19–13.07	0.024^b^
GG	1/0	—	—	—^c^
AG + GG genotypes	9/10	4.15	1.28–13.50	0.018^b^
rs3842787 (C > T)				
CT	10/15	2.42	0.81–7.21	0.110
TT	1/0	—	—	—^c^
CT + TT genotypes	11/15	2.56	0.88–7.44	0.084
rs5788 (C > A)				
CA	18/33	1.83	0.76–4.39	0.176
AA	3/8	1.58	0.33–7.42	0.558
CA + AA genotypes	21/41	1.77	0.81–3.86	0.151
*NOS3* gene				
rs2070744 (C > T)				
CT	27/39	4.00	1.84–8.71	<0.001^b^
TT	16/30	3.20	1.29–7.92	0.012^b^
CT + TT genotypes	43/69	3.66	1.90–7.04	<0.001^b^
rs1799983 (G > T)				
GT	26/29	4.54	2.10–9.82	<0.001^b^
TT	2/9	2.15	0.30–15.43	0.445
GT + TT genotypes	28/38	4.21	2.00–8.89	<0.001^b^
NSAID users^d^				
*PTGS1* gene				
rs1330344 (C > T)				
CT	17/30	5.74	2.56–12.85	0.024^b^
TT	15/35	2.47	1.11–5.50	0.027^b^
CT + TT genotypes	32/65	3.70	2.05–6.66	<0.001^b^
rs10306114 (A > G)				
AG	5/8	5.69	1.46–22—07	0.012^b^
GG	0/0	—	—	—^c^
AG + GG genotypes	—	—	—	—
rs3842787 (C > T)				
CT	8/13	5.69	1.86–17.39	0.002^b^
TT	0/0	—	—	—^c^
CT + TT genotypes	—	—	—	—
rs5788 (C > A)				
CA	12/23	2.38	0.98–5.77	0.055
AA	4/5	4.21	0.84–21.03	0.079
CA + AA genotypes	16/28	2.71	1.25–5.88	0.012^b^
*NOS3* gene				
rs2070744 (C > T)				
CT	15/35	2.50	1.13–5.54	0.024^b^
TT	16/15	10.99	4.25–28.38	<0.001
CT + TT genotypes	31/50	4.43	2.37–8.26	<0.001^b^
rs1799983 (G > T)				
GT	17/23	4.57	2.01–10.37	<0.001^b^
TT	5/4	37.07	5.67–242.21	<0.001^b^
GT + TT genotypes	22/27	6.53	3.10–13.74	<0.001^b^

N, number of participants of case/control groups; OR, odds ratio; CI 95%, confidence interval 95%; LDA, low-dose aspirin; NSAIDs, nonsteroidal anti-inflammatory drugs.

a Analysis adjusted to ethnicity; body mass index; history of ulcer, bleeding and dyspepsia; cardiovascular, blood, and respiratory diseases; *Helicobacter pylori* serology; reliability of the interview; use of oral anticoagulants and nonsteroidal anti-inflammatory drugs; smoking habit; alcohol intake; and amount of coffee consumption per day.

b Statistical significance.

c It was not possible to conduct the analysis with the homozygous category for variant allele due to the absence of participants or reduced sample size.

d Analysis adjusted to ethnicity; body mass index; history of ulcer, bleeding and dyspepsia; cardiovascular, blood and respiratory disease; *Helicobacter pylori* serology; reliability of the interview; use of oral anticoagulants and low-dose aspirin; smoking habit; alcohol intake; and amount of coffee consumption per day.

The category of reference for genetic variants is the wild phenotype.

Regarding the *NOS3* gene, LDA users showed an UGIB risk about four times higher in those carriers of the CT + TT genotypes *vs.* CC of rs2070744 (OR: 3.66, CI 95%: 1.90–7.04) and in carriers of the GT + TT genotypes *vs.* GG of rs1799983 (OR: 4.21, CI 95%: 2.00–8.59). A higher risk of UGIB was observed in NSAIDs users than in LDA users: CT + TT genotypes *vs.* CC of rs2070744 (OR: 4.43, CI 95%: 2.37–8.26) and carriers of the GT + TT genotypes *vs.* GG of rs1799983 (OR: 6.53, CI 95%: 3.10–13.44), respectively (Table 4).

In the logistic regression model, personal history of ulcer (OR: 3.82, CI 95%: 1.88–7.75); *Helicobacter pylori* infection (OR: 1.91, CI 95%: 1.16–3.16); LDA (OR: 3.70, CI 95%: 1.88–7.27), NSAIDs (OR: 4.66, CI 95%: 2.57–8.43), and oral anticoagulant use (OR: 10.58, CI 95%: 3.58–31.25); and alcohol intake (0 < consume ≤30 g of alcohol/day: OR: 2.58, CI 95%: 1.41–4.74 and >30 g of alcohol/day: 7.98, CI 95%: 2.98–21.32) were identified as independent risk factors for UGIB (Table 3).

Personal history of dyspepsia (OR: 0.40, CI 95%: 0.24–0.69), overweight (OR: 0.54, CI 95%: 0.33–0.89), blood (OR: 0.14, CI 95%: 0.02–0.81) and respiratory disease (OR: 0.18, CI 95%: 0.05–0.57), and dyslipidemia (OR: 0.21, CI 95%: 0.10–0.46) were identified as protection factors for UGIB. Heterogeneity in reliability of the interview was identified between the case and control groups (chi-square < 0.001—Table 1), and this variable showed statistical significance in the logistic regression model when scores were eight and nine (OR: 0.57, CI 95%: 0.34–0.95) (Table 3).

## Discussion

This is an unprecedented study in the Brazilian population, and it assessed the influence of six genetic variants in the *PTGS1* (rs1036114, rs3842787, rs1330344, and rs5788) and *NOS3* genes (rs2070744 and rs1799983) on the risk of UGIB.

The four genetic variants evaluated in the *PTGS1* gene were associated with an increased risk of UGIB. Despite a wide clinical review that reported genetic variants in this gene to appear more associated with the prognosis of a disease than with the risk of developing it (Agúndez et al., 2015), it has also been suggested that carriers of these genetic variants are at an increased risk of bleeding (Halushka et al., 2003; Maree et al., 2005). Indeed, carriers of variants rs10306114 and rs3842787 may have a *COX-1* with less capacity to produce prostaglandins, which can also enhance sensitivity to LDA and NSAIDs (Halushka et al., 2003), and carriers of variants rs1330344 and rs5788 may show a reduction in the platelet aggregation and, consequently, an increased risk of bleeding (Maree et al., 2005).

Except for rs1036114, which is an unprecedented analysis, three other studies assessed the influence of rs3842787, rs1330344, and rs5788 on the UGIB risk (van Oijen et al., 2006; Wu et al., 2016; Mallah et al., 2020). In line with our findings, Mallah and colleagues suggested that rs1330344 was associated with an increased risk of UGIB in LDA users (Mallah et al., 2020), and Wu and colleagues reported rs1330344 as a possible risk factor for the gastric mucosal injury induced by LDA (Wu et al., 2016),whereas two studies did not identify association between genetic variants rs3842787 and rs5788 and UGIB risk (van Oijen et al., 2006; Wu et al., 2016).

Comparing our findings with those of these three studies, it is important to highlight that despite the enriching clinical discussion carried out by van Oijen et al*.* (2006) and Wu et al. (2016), there are important methodological limitations (van Oijen et al., 2006; Wu et al., 2016). In the study by van Oijen et al., there is a lack of clarity in the recruitment and matching process, in addition to a small sample size (106 cases and 74 controls), while in the study by Wu et al., aspects of unmatched participants, not adjusting to confounding variables, and inclusion of only 13 participants in the UGIB group were observed. From another perspective, besides the methodological aspects, the epidemiological profile and population miscegenation of the included participants should be considered. It is well known that the Brazilian population is highly mixed, which will directly influence our findings, since ethnicity and ancestry markers influence the presence and frequency of genetic variants in populations (Ramos et al., 2014; Rodrigues-Soares et al., 2018).

Regarding the *NOS3* gene, the SNPs rs2070744 and rs1799983 have been widely studied as possible risk factors for cardiovascular diseases (Zhang et al., 2012; Nawaz et al., 2015), and rs1799983 was identified as a genetic biomarker of coronary artery disease in a meta-analysis of 132 case–control studies (Li et al., 2019). Meanwhile, little is known regarding the presence of these variants in the pathophysiology of UGIB.

Despite several studies that evaluated the influence of genetic variants in the idiosyncratic response to LDA treatment (Shiotani et al., 2014, 2015; Wang, 2019) and also in interindividual differences in platelet activation in LDA users (Postula et al., 2011), to our knowledge, only one study assessed the rs1799983 within the scope of UGIB (Mallah et al., 2020). Mallah et al. found an increased risk of UGIB in the presence of genetic variation in LDA users (OR: 7.69, CI 95%: 2.40–23.7, *p*-value: < 0.001), corroborating with our findings (OR: 4.21, CI 95% 2.00–8.89, *p*-value: 0.001). The wide confidence interval observed in the study by Mallah et al. may be justified by the low sample size of participants included in the analysis (nine cases and five controls) when compared to the sample of our study (28 cases and 38 controls).

One hypothesis for the identified increased risk of UGIB in LDA users would be that the presence of the genetic variant rs1799983 influences the expression of *NOS3* gene activity and affects serum nitrate levels, which compromises important physiological mechanisms (e.g., gastric protection and platelet aggregation) (Lanas, 2008; Vecoli, 2014). Therefore, the compromise of these mechanisms, in addition to possible gastrointestinal damage associated with LDA use (Lanas et al., 2015), may be a clinical rationale for the increased risk of gastrointestinal bleeding.

Considering LDA is widely prescribed in the prevention of cardiovascular events, an increase on UGIB risk can be an important healthcare issue. In addition, despite well-established benefits of LDA in secondary prevention (Parekh et al., 2013), the risks can outweigh the unclear benefits (e.g., reduction of mortality or incidence of new cardiovascular events) in primary prevention (Collins et al., 2009), especially due to the risks of bleeding (Kochar and Gaziano, 2010). Furthermore, although several studies have been assessing the use of LDA in cancer prevention, a systematic review that included 103,787 participants did not identify a reduction in the incidence of cancer or in the overall cancer mortality (Chubak et al., 2016).

In this context, individualized assessment of the risk/benefit of LDA and NSAIDs use, treatment monitoring, and development of guidelines for the evaluation of genetic variants potentially involved in idiosyncratic responses may be effective strategies in the risk management. In NSAIDs and warfarin users, for instance, international guidelines already recommend the assessment of genetic variants involved in the metabolism of these drugs and potentially associated with possible safety risks (e.g., *CYP2C9* and *VKOCR1*) (Johnson et al., 2011; Swen et al., 2011). Besides, an effective risk communication to improve signal detection of possible adverse drug events related to LDA use can help reduce the harm of medication. However, in Brazil, despite efforts to harmonize pharmacovigilance regulations with the world, several concerns remain, particularly those that impair effective risk communication and signal detection (e.g., a lack of awareness or incentive to report patient effects and underreporting) (Varallo et al., 2019).

It is important to highlight that in addition an increased risk of UGIB was also identified with other drug class use (e.g., oral anticoagulants), *Helicobacter pylori* infection, and lifestyle habits (e.g., alcohol intake). Hence, to identify patients at higher risk for UGIB or rebleeding, a clinical care pathway for the management of the patients diagnosed with UGIB is needed (Franco et al., 2015). Furthermore, studies have been documenting the effectiveness of the prescription of proton pump inhibitors in patients on LDA therapy or with high risk of UGIB (Lanas et al., 2011) as well as eradication of *Helicobacter pylori* (Lanas and Scheiman, 2007).

Finally, our study has strengths and limitations. To our knowledge, this is the first Brazilian study to assess genetic variants of the *PTGS1* and *NOS3* genes within the scope of the UGIB, in addition to unprecedented analysis of two SNPs (rs10306114 and rs2070744). The following can be mentioned as strengths of this study: the adjusted analysis for well-documented variables for UGIB risk (e.g., NSAIDs, oral anticoagulant, and LDA use; *Helicobacter pylori* infection; and lifestyle habits), inclusion of only biologically unrelated patients in order to reduce possible biases, and data collection through face-to-face interviews. The limitations of this study are the impossibility to analyze the influence of carrying more than one SNP and the risk of UGIB in addition to the reduced sample size of LDA and NSAIDs users.

In summary, this study presents two unprecedented analyses within the scope of the UGIB (rs10306114 and rs2070744), and our findings suggested an increased risk of UGIB in the presence of the variants rs10306114 and rs5788, regardless of the drug exposure. Besides, the presence of the evaluated genetic variants might modify the magnitude of the risk of UGIB in LDA/NSAIDs users. Hence, patients undergoing chronic LDA and NSAIDs treatment should be monitored in order to reduce harmful medication use, and our findings can contribute with evidence to the scope of pharmacogenomics and personalized therapy, promoting patient safety.

## Data Availability

The raw data supporting the conclusions of this article will be made available by the authors, without undue reservation, to any qualified researcher.
